# Assessment of a novel cryoablation device for the endovascular treatment of cardiac tachyarrhythmias

**DOI:** 10.1177/2050312118769797

**Published:** 2018-05-04

**Authors:** John M Baust, Anthony Robilotto, Peter Guerra, Kristi K Snyder, Robert G Van Buskirk, Marc Dubuc, John G Baust

**Affiliations:** 1 CPSI Biotech, Owego, NY, USA; 2 Institute of Biomedical Technology, The State University of New York, Binghamton, NY, USA; 3 Department of Biological Sciences, Binghamton University, Binghamton, NY, USA; 4 Montreal Heart Institute, Montreal, QC, Canada

**Keywords:** Cryoablation, supercritical nitrogen, atrial fibrillation, catheter ablation

## Abstract

**Objectives:**

Cryoablation is an effective alternative treatment for cardiac arrhythmias offering shortened recovery and reduced side effects. As the use of cryoablation increases, the need for new devices and procedures has emerged. This has been driven by technological limitations including lengthy periods to generate a single lesion (3–5 min), uncertain transmurality, and differential efficacy. Furthermore, due to limited ablation capacity under high heat loads, cryo has had limited success in the treatment of ventricular arrhythmias. To this end, in this study we evaluated a new cryoablation catheter, *ICEolate*, for the targeted ablation of cardiac tissue.

**Methods:**

Performance assessment included calorimetry, freeze zone isothermal distribution characterization and catheter ablation capacity in a submerged, circulating, heat-loaded ex vivo tissue model. A pilot in vivo study was also conducted to assess ablative capacity of the cryocatheter in a fully beating heart.

**Results:**

Ex vivo studies demonstrated ice formation at the tip of a cryocatheter within 5 s and a tip temperature of ~−150°C within 10 s. The device repeatedly generated freeze zones of 2 cm × 3 cm in less than 2 min. Tissue model studies revealed the generation of a full thickness (5–10 mm) cryogenic lesion within 1 min with an opposite (transmural) surface temperature of <−60°C under a circulating 37°C heat load. Pilot in vivo studies demonstrated the delivery of an ablative “dose,” producing a continuous full thickness transmural linear lesion in <60 s at both atrial and ventricular sites.

**Conclusion:**

These studies suggest that the supercritical nitrogen cryodevice and *ICEolate* cryocatheter may provide for rapid, effective, controllable freezing of targeted tissue. The ablative power, speed, and directional freeze characteristics also offer the potential of improved safety via a reduction in procedural time compared to current cryoablation devices. These technological developments may open new avenues for the application of cryo to treat other cardiac arrhythmogenic disorders.

## Introduction

Cryotherapy (cryo) is an effective, minimally invasive alternative to surgery and heat-based therapies offering patients a shortened recovery and reduced side effects.^1–3^ A plethora of studies have detailed the use of various cryosurgical devices and procedures to apply freezing temperatures to a target tissue.^4–12^ With the recent reports on the success of cryo for atrial fibrillation (AF) treatment, there is now increasing use of cryo as a standalone, minimally invasive procedure to treat many forms of cardiac arrhythmias.^4,13–18^ As the use of cryotherapy continues to grow, improvements to device design and application will further increase its utilization for the treatment of cardiac arrhythmias.^4–12,19,20^

With the recent success of cardiac cryoablation, there is now a movement toward the increasing indication utilization and improving procedure outcome. In order to achieve this in an effective, time-efficient manner, new cryotechnologies must be developed to facilitate rapid tissue cooling to more effectively treat cardiac arrhythmias. The aim of this study was to investigate the performance of a new supercritical nitrogen (SCN)-based *ICEolate* cryocatheter for utilization for the endovascular cryoablation of cardiac tissue. One objective of our cryogenic technology development activities is to enable the rapid delivery of ultra-cold ablative temperatures to cardiac tissue in a minimally invasive, endovascular catheter-based format, thereby creating a technological path for cryo to potentially become a primary treatment option for expanded cardiac arrhythmia indications, including ventricular tachyarrhythmias (VT). We hypothesized that through the use of SCN and catheter technologies, that rapid, effective, minimally invasive ablation of cardiac tissue could be achieved via catheter ablation (CA). If effective, such a technology could provide a platform enabling the delivery of a precise, minimally invasive cryoablation treatment option for VT, thereby reducing overall procedural outcome variability, cost, recovery time, and associated morbidity. In order to assess the potential of this next generation cryogen platform, in this study, we conducted a series of ex vivo studies and a pilot in vivo study to characterize and evaluate the performance of *ICEolate* for endovascular catheter based cardiac cryoablation.

## Methods

### SCN system and *ICEolate* cryocatheter

All tests were performed using the SCN cryoablation device with an initial cryogen pressure of 1300 ± 100 lbf/in^2^ in conjunction with the *ICEolate* cryocatheter. The *ICEolate* endovascular catheter design consisted of a 1-m-long 10F catheter with a 15-mm blunt tip ablation segment at the distal end. The *ICEolate* cryocatheter was connected to the SCN cryoconsole via a 3-m umbilical. For analysis, the cryocatheter was positioned in the respective test fixture containing medium and then activated for a freeze time of 120 or 180 s (2 or 3 min; Assay dependent, see details of specific testing below).

### Calorimetry assessment

**Figure 1.**
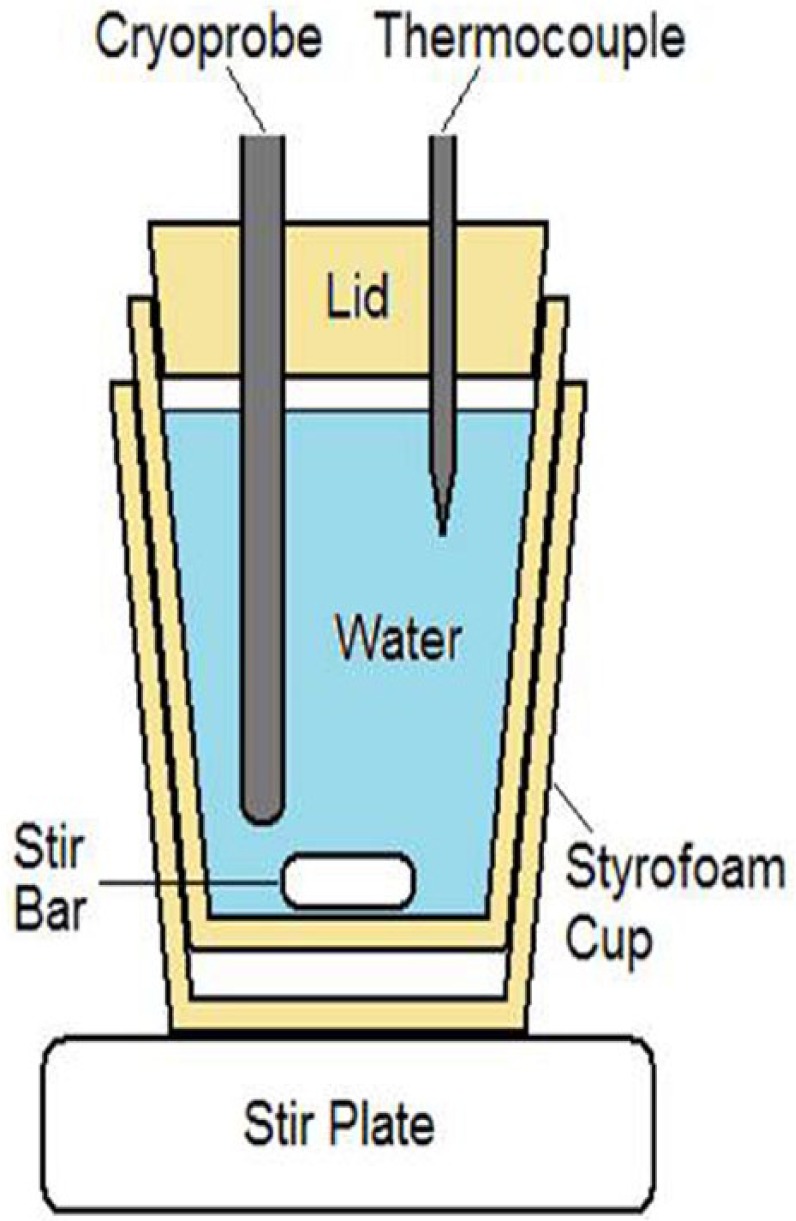
Illustration of the calorimetry testing fixture. The setup is designed to prevent ice formation on the probe surface during a freeze and allows for assessment of temperature change of water during a given freeze run. Time and temperature data are then utilized to calculate the cooling power of a given cryoprobe.

Calorimetry testing was conducted as follows: a 30-mm t-style magnetic stir-bar was placed on the bottom of a 20 oz double-walled foam-insulated vessel (styrofoam cup within a cup). The insulated vessel was filled with 454-mL of water, and a ~20-mm-thick polystyrene lid was placed on top for added insulation. The setup was placed on a magnetic stir plate, and the speed was set to the highest setting in which the stir-bar maintained uniform spinning. The cryocatheter was placed through a tight fitting hole in the lid with the distal end 20 mm off the bottom of the vessel (to ensure that the entire freeze zone was submerged but did not contact the stir bar or the vessel wall). A 21-ga (0.032 in) type-T thermocouple needle was inserted through the lid into the water to monitor the temperature change. A 3-min freeze procedure was performed, and the starting and ending water temperatures were recorded. An illustration of the calorimeter setup is presented in Figure 1.

Cooling power was determined using standard calorimetry calculations

$$P=\frac{\Delta Q}{\Delta t}=\frac{m_{w}\cdot s_{w}\cdot \Delta T}{\Delta t}$$

where *P* is the cooling power, *Q* is the heat energy, *m_w_* is the mass of water, *s_w_* is the specific heat of water (4.186 J/g°C), *T* is the temperature, and *t* is the time (length of freeze procedure).

### Freeze zone size and thermal profile assessment

#### Iceball diameters

**Figure 2.**
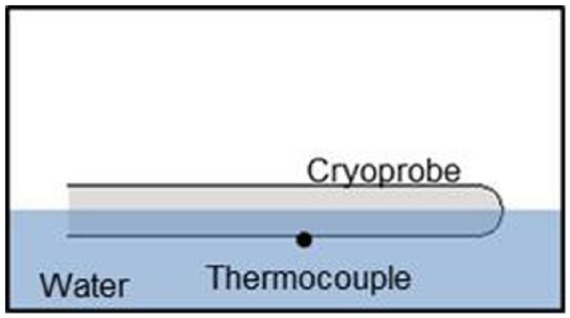
Illustration of the partially submerged cryocatheter testing setup. The fixture allows for visualization of iceball formation and measurement of overall freeze zone size during and following a given freeze interval. The thermocouple positioned on the exterior surface allows for the assessment of probe surface temperature under various heat loads.

*Partially submerged ablation segment*: an acrylic testing fixture (105 mm × 125 mm × 55 mm) was filled with room temperature (20°C ± 2°C) water, and the cryocatheter was laid horizontally on the surface (Figure 2). The probe height was adjusted such that half of the ablation segment probe (1.7 mm) was submerged, and a 120-s freeze was performed. Following the freeze procedure, the iceball diameter was measured at the midpoint of the ablation segment. The time to first ice was also recorded.

*Fully submerged ablation segment*: cryocatheters were placed vertically into the center of an acrylic testing fixture (51 mm × 51 mm × 200 mm) filled with room temperature (20°C ± 2°C) water to a level ~10 mm above the proximal end of the freeze zone. A 120 s freeze was performed, and the iceball diameter was measured at the middle of the ablation segment (7.5 mm from the tip).

#### Thermal profile of freeze zone

*Cryoprobe surface temperature*: for assessment of the cryocatheter external surface temperature, a 36-ga type-T thermocouple was affixed at the midpoint of the freeze zone (Figure 2). The cryocatheter and thermocouple were placed horizontally into an acrylic testing fixture (105 mm × 125 mm × 55 mm) filled with room temperature (20°C ± 2°C) water. The probe was adjusted such that half of the probe (1.7 mm) was submerged, and the thermocouple was at the bottom of the probe (submerged in the water). A 180-s freeze was performed, and temperatures were recorded at a rate of 1/s.

**Figure 3.**
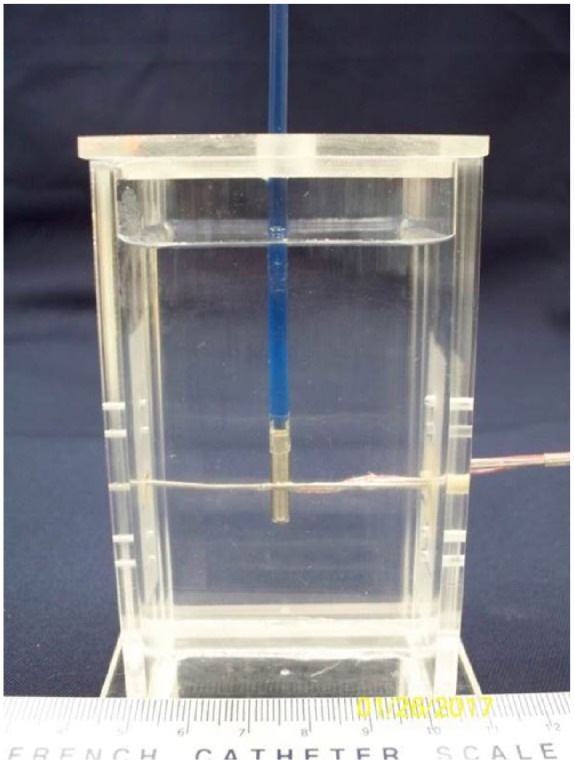
Image of the fully submerged isothermal profile assessment fixture. The fixture contains a water or ultrasound gel medium and a mandrel with thermocouples affixed at five pre-set distances allowing for real-time recording of a freeze run and determination of isotherm location at any point during a given freeze.

*Thermal profiles of iceballs*: the cryocatheter ablation segment was placed into the center of an acrylic testing fixture (51 mm × 51 mm × 200 mm) filled with room temperature (20°C ± 2°C) ultrasound gel to a level ~10 mm above the proximal end of the probe freeze zone (Figure 3). A thermocouple mandrill consisting of five 36-ga type-T thermocouples soldered to a 22XT (0.7-mm OD) stainless steel tube was then placed at the midpoint of the freeze zone.^21^ The thermocouples and mandrill were situated such that temperature recordings were taken at 1, 6, 11, 13.5, and 16 mm extending radially from the cryoprobe surface. Temperatures were recorded at a rate of 1/s, and all tests were run for 180 s.

#### Freeze repeatability testing

For assessment of cryocatheter freeze repeatability, the cryocatheter was placed into a warm water bath and activated for four consecutive 15-s or 30-s freezes with a 30-s thaw cycle between each freeze. As with thermal profile testing, probe surface temperature was monitored via a type-T thermocouple placed within the freeze chamber of the cryocatheter ablation segment.

### Ex vivo endocardial tissue analog

**Figure 4.**
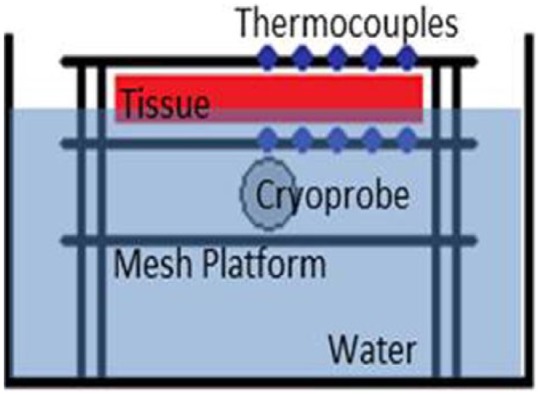
Illustration of the heat loaded ex vivo tissue isotherm assessment setup. The fixture allows for real-time assessment of thermal profiles within the tissue both on the proximal and transmural surface of the tissue section.

Ex vivo endocardial tissue model testing consisted of a cryocatheter placed into a circulating 37°C water bath and a 5- or 10-mm thick piece of porcine skeletal muscle tissue placed on top of the catheter within the bath. Thermocouples were placed on the proximal and distal surfaces of the tissue to monitor tissue temperature during an ablation interval (Figure 4). The specific setup consisted of a vinyl mesh platform, without thermocouples, placed into the 175 mm × 140 mm × 90 mm acrylic box, and the cryoprobe was laid on top. A second mesh platform, with an identical type-T thermocouple layout as in the thermal profile testing above, was then placed atop the cryoprobe. The acrylic box was filled with 37°C ± 2°C water to a level ~1 mm above the upper mesh platform, and a section of bovine skeletal muscle, ~100 mm × 45 mm × 4.5–5 mm, was placed on top of the upper mesh platform. Finally, a third mesh platform, also containing an identical type-T thermocouple layout, was placed atop the tissue section to monitor the transmural surface (Figure 4). A second series of tissue studies was also conducted using a 1-cm-thick section of porcine muscle (~100 mm × 45 mm × 10 mm). The water in the fixture was maintained at 37°C and was circulated at a rate of 4.5 L/min using a rotary pump during the 180 s (3 min) freeze procedure.

### Pilot endocardial in vivo study

In vivo testing was completed in an acute canine model under the supervision of Dr Guerra at the Montreal Heart Institute. Cryoablation targeting right atrial and ventricle tissue was performed using a first-generation prototype blunt-tip, non-deflectable *ICEolate* catheter. Three mongrel canines (<6 years of age and between 35–40 kg) were utilized, and 3–4 ablations were performed in each animal (10 ablations total) in this pilot study. The objective of the pilot study was to assess the functionality of the prototype SCN *ICEolate* catheter (i.e. maneuverability, visualization, ability to deliver site directed tissue ablation, freeze time and temperature, and electrical block). The combination of three animals and three to four treatment sites per animal provided for 10 ablation sites for analysis of procedural outcome and allow for statistical assessment (*n* = 10). The sample size for this study was model after previous pilot studies, and study sample size was calculated using G Power (correlation: point biserial model—a priori: effect = 0.6, *α*-error = 0.2, power = 0.8), which yielded a minimum sample size of *n* = 6 for the pilot study. The sample size of 10 was selected to allow for sample attrition without compromising significance. Studies were conducted under the guidance and approval of the Montreal Heart Institute Committee of Animal Ethics. All animals used in this project were treated with compassion, according to the principles of the Canadian Council for the protection of animals such as described in the Manual on the Care and Use of Experimental Animals. Animals were tranquilized, sedated, intubated, and anesthesia was maintained throughout the procedure with vital signs monitored on electrocardiogram (ECG). Femoral and jugular veins provided endocardial catheter access to the heart and hemodynamic values were monitored via femoral artery access. The NAVEX system was utilized for three-dimensional (3D) mapping of the heart. Cryolesions were created using the *ICEolate* cryocatheter with cryoapplications of 15, 30 and 60 s. Electrical activity at the target site was monitored prior to, during and following freezing via unipolar ECG at the catheter tip. Following the procedure, animals were euthanized by increasing the anesthetic to 5% followed by an injection of KCl (20 mEq/10 mL). Hearts were then explanted, prepared and gross pathological analysis was performed.

### Data analysis

For calorimetry, iceball, thermal profile assessment, and ex vivo tissue analog studies, a minimum of three repeats per probe were conducted. Following experimentation, data were combined and averaged (± standard deviation (SD)) to determine mean iceball size, temperature location, and ablation zone produced. Statistical significance was determined using single factor analysis of variance (ANOVA) where noted. Freeze zone size: following the freeze/thaw episode, iceball diameter was measured using digital calipers at the center point of the cryoprobe freeze zone. Thermal Profiles: real-time recordings of the thermal profiles were collected using an Omega TempScan system and were converted to graphical format using Microsoft Excel and analyzed to determine isotherm spread during the freeze intervals. All temperatures were recorded at a rate of 1/s.

## Results

### Calorimetry testing

Evaluations of cryocatheter cooling power were conducted using a calorimetry testing setup. Initial testing was conducted using a starting water temperature of 35°C; however, it was found that due to the increased cooling capacity of the SCN *ICEolate* cryocatheter, this was not able to prevent ice formation on the surface of the cryoprobe regardless of the level of stirring of the water bath. Prevention of ice formation along the freeze zone during calorimetry testing is important as ice along the probe shaft serves has an insulative effect decreasing heat extraction capacity, thereby yielding inaccurate (suppressed) readings. As such, the water temperature was increased until minimal visual ice was apparent on the probe surface during the test run. This resulted in a final starting bath temperature of 85°C. Once the bath temperature was determined, calorimetric assessment of the cryocatheter was conducted (Table 1). Assessment of the 10F endovascular cryocatheter revealed an average decrease in temperature of 12°C (±0.8) yielding an average cooling power of 126.7 W (±9.2) and yielding a power output/mm for the catheter of 8.4 W/mm (± 0.6).

**Table 1.** Average calorimetry data for SCN *ICEolate* cardiac cryocatheter following a 2-min freeze.

Probe	Water temperature (°C)			Watts
	Start	End	Δ*T*	
Catheter	86.4	74.4	12.0	126.70

### Freeze zone size and thermal profile assessment

#### Freeze zone size

Evaluation of freeze zone (iceball) size were conducted using two heat load scenarios, partial and full submersion. These scenarios were designed to evaluate the relative impact of increased heat load on cryoprobe performance. Analysis of time to initial ice formation (visualization of ice on the ablation segment) revealed no substantial difference between the partial and fully submerged scenario with the cryocatheter achieving initial ice formation in less than 5 s following activation (Table 2). Assessment of freeze zone size also revealed the attainment of a similar diameter and length iceball. Specifically, an average iceball diameter of ~2.0 cm with a length of ~3.2 cm was attained following a 120-s (2-min) freeze in both settings (Table 2).

**Table 2.** Ice kinetics of a partially and fully submerged catheter following a 2-min freeze.

Test setup	Time to ice (s)	Iceball diameter (cm)	
		Width	Length
Partial	1.7	1.93	3.17
Full	1.3	2.10	3.20

#### Thermal profile of freeze zone

**Figure 5.**
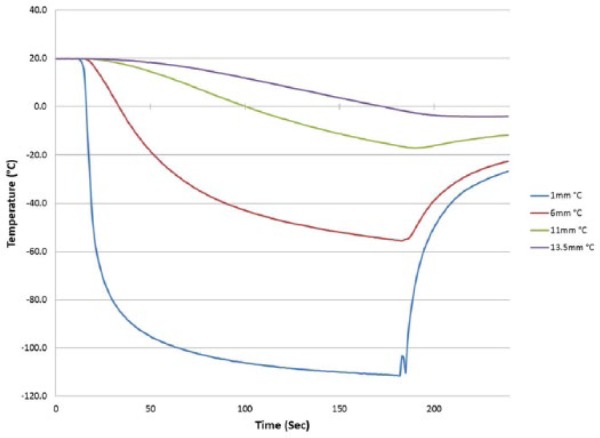
Real-time monitoring of the isothermal profile generated by the *ICEolate* cryocatheter at the center of the ablation segment during a 3-min freezing protocol. Temperatures of the mid-point of the freeze zone were monitored at fixed points radiating from the cryoprobe surface in real-time at 1-s intervals during a 3-min freeze procedure. Temperatures were found to drop quickly near the probe surface and more gradually the further from the probe surface. Thermal profile assessment revealed a final penetration of the −20°C, −30°C, and −40°C isotherms to reach diameter of 2.1, 1.9, and 1.7 cm, respectively. Analysis of the thermal profiles revealed that on average 39% and 19% of the frozen mass was encompassed within the −20°C and −40°C isotherms, respectively.

Following the establishment of freeze zone size, analysis of the thermal profile within the iceball was conducted. This included assessment of ablation segment surface temperature as well as monitoring temperature distribution outward from the probe surface. Temperatures were recorded in real time at 1-s intervals at the probe surface, 1, 6, 11, 13.5 and 16 mm radially from the probe during a 180 s (3 min) freeze (Figure 5). Following completion of the freeze, the depth of penetration of the −20°C, −30°C, and −40°C critical isotherms was determined. Analysis of surface temperature revealed the attainment of a nadir temperature of −149.4°C (Table 3). Importantly, a probe surface temperature of −100°C was attained within 5 s of probe activation (Figure 5(c)). Analysis of the isothermal distribution within the frozen mass revealed that the −20°C isotherm extended a diameter of 2.1 cm from the catheter surface, the −30°C isotherm extended 1.93 cm, and the −40°C isotherm extended 1.72 cm from the surface. Volumetric analyses for the catheter assumed a spherical shape, revealed a frozen mass of 4.8 and 6.8 cm^3^ after a 3-min freeze. Calculating the volume of ice contained at or below each critical isotherm revealed that 72% (4.8 cm^3^) of the total frozen volume was below −20°C, 56% (3.8 cm^3^) ≤ −30°C and 39% (2.7 cm^3^) ≤ −40°C.

**Table 3.** Thermal profile of a fully submerged *ICEolate* cryocatheter.

Time to ice (s)	Nadir surface temperature (°C)	Time to −100°C	Iceball diameter (cm)		Isotherm diameter at 3 min (cm)		
			2 min	3 min	−20°C	−30°C	−40°C
2.0	−149.4	5 s	2.10	2.35	2.10	1.93	1.72

#### Freeze repeatability testing

**Figure 6.**
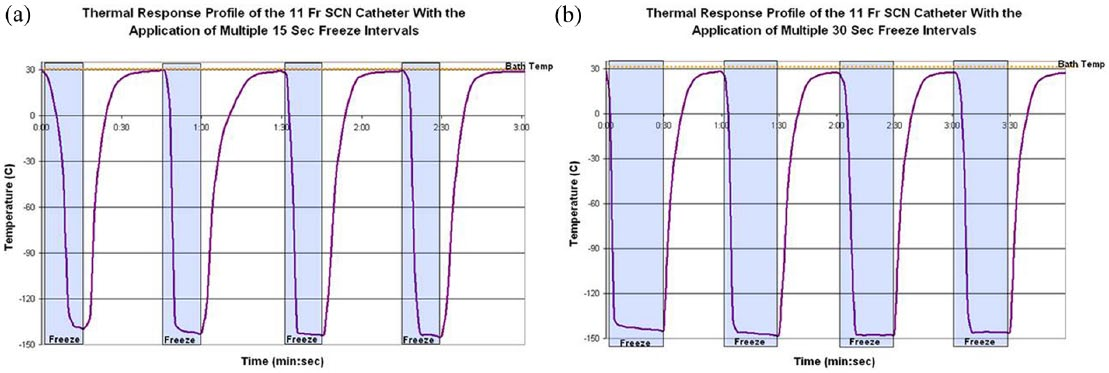
Thermal profiles of the cryoprobes during repeated 15-s and 30-s freeze cycling. The *ICEolate* cryocatheter (1.5-cm freeze zone/110-cm catheter/2-m umbilical) was placed into a warm water bath and repeatedly activated for (a) 15-s or (b) 30-s freeze intervals with a 30-s thaw between each of the four cycles. The cryocatheter (entire catheter submerged) yielded a thermal profile of ice formation in <5 s and achieved a temperature of −145°C (± 4°C) within 10 s.

One critical aspect of device performance for application in a cardiac setting is that of quick and consistent application of freeze ablation. In order to test the utility of the *ICEolate* cryocatheter under this scenario, a repeat freeze challenge model was utilized. To this end, the cryocatheter was placed into a heated water bath (30°C) and cryogen flow was cycled for four consecutive freezes of 15 and 30 s with a 30-s pause between each freeze. Probe surface temperature was monitored throughout the cycling process. In addition, time to nadir temperature and the ability of the temperature to return to starting temperature within the thaw window were also monitored (Figure 6). Cycling analysis revealed that the *ICEolate* cryocatheter was able to rapidly and repeatedly achieve a nadir probe surface temperature of −145°C (±4) in <5 (±1) s and was able to completely rebound (return to starting temperature) following ending of cryogen flow within the 30-s thaw window. Importantly, upon stopping of the SCN cryogen flow to the probe, the probe started warming in <1 s demonstrating, practically speaking, instantaneous and repeatable on/off control of the freezing event.

### Ex vivo tissue analog testing

#### Endocardial analog testing

**Figure 7.**
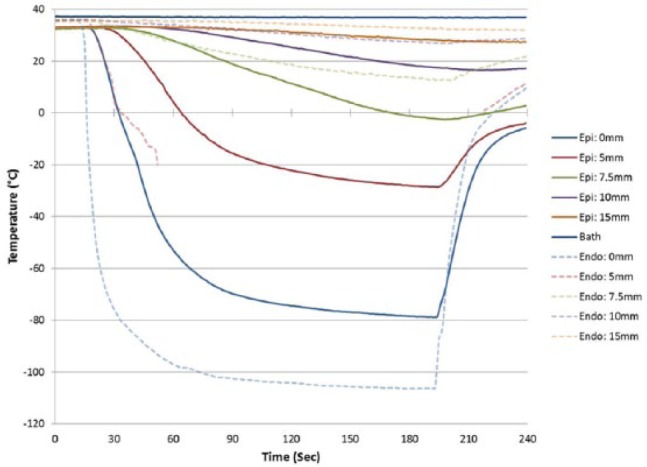
Real-time monitoring of the isothermal profile generated by the *ICEolate* cryocatheter in the heat load ex vivo bovine tissue model 3-min freezing protocol. Temperatures of the mid-point of the freeze zone were monitored at fixed points radiating from the cryoprobe surface on both the proximal (endocardial) and distal (epicardial) tissue surface. Temperatures were recorded in real-time at 1-s intervals during a 3-min freeze procedure. Temperatures were found to drop quickly near the probe surface and more gradually the further from the probe surface on the proximal (endocardial) surface. Temperatures on the distal surface (epicardial) decreased rapidly directly above the cryoprobe achieving <−30°C across the entire tissue thickness within 45 s.

**Figure 8.**
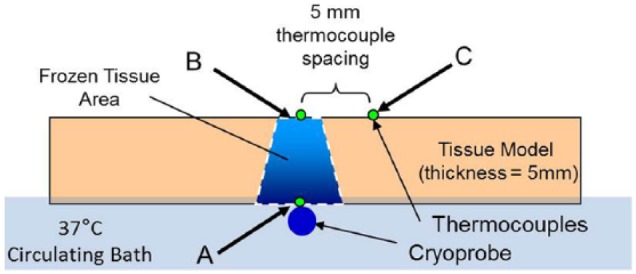
Illustration of the freeze zone and thermal spread generated by the *ICEolate* cryocatheter in the heat load ex vivo bovine tissue model freezing protocol. Temperatures of the mid-point of the freeze zone were monitored at fixed points radiating from the cryoprobe surface on both the proximal (endocardial) and distal (epicardial) tissue surface. Temperatures were found to drop below −80°C at the probe tissue interface within 30 s (a). Temperatures on the distal surface (epicardial) decreased rapidly directly above the cryoprobe (midline) achieving <−30°C across the entire tissue thickness within 45 s (b). Importantly, delivery of the ablative dose across the tissue thickness was found to be highly precise with a tissue temperature of +12°C recorded 5 mm from the midline on the distal surface (c) when the midline point (b) reached −30°C.

With establishment of the freeze zone dimensions following a 120-s (2-min) freeze in a partial heat load model, studies were conducted to assess *ICEolate* cryocatheter performance under full heat load. To this end, the cryocatheter was placed within in a circulating (4.5 L/min) 37°C water bath, then a section of bovine cardiac tissue was placed on top of the cryocatheter partially submerged within the circulating bath (Figure 4). An array of thermocouples was positioned radiating perpendicular to the ablation segment at the catheter tissue interface as well as on the opposite surface of the tissue to allow for thermal profile monitoring throughout the freeze process (Figure 7). Analysis revealed the creation of a 1.86-cm-diameter freeze zone on the opposite/transmural surface (epicardial surface) of a 5-mm-thick tissue section following a 180-s (3-min) freeze under full heat load using the *ICEolate* cryocatheter. Analyses of the thermal profile of the opposite/ transmural (epicardial) surface revealed that a temperature of 0°C was achieved in 32 s, −20°C in 45.0 s, and −30°C in 52.0 s directly above the probe tissue interface (Table 4). The nadir transmural tissue surface temperature recorded was −67.6°C under full circulation heat load. The thermal spread of the ablation zone revealed the temperature 5-mm perpendicular to the center line of the freeze zone when the transmural 0-mm position (directly above the ablation segment) reached −30°C, was 12.8°C on the transmural (epicardial) surface and −9.1°C on the endocardial (probe side) surface (Figure 8). This suggested that the *ICEolate* cryocatheter was able to deliver an ablative thermal dose of −30°C across a 5-mm tissue segment under full heat load in <60 s while resulting in minimal unwanted destructive thermal spread within the tissue.

**Table 4.** Thermal profile of the catheter on an endocardial analog.

Tissue thickness (mm)	Iceball diameter (cm)	Time to transmural temperature (s)			Nadir transmural temperature (°C)	Transmural thermal spread at 5 mm (°C)
		0°C	−20°C	−30°C		
4.5	1.86	31.7	45.0	52.0	–67.6	12.8

### Pilot endocardial in vivo study

**Figure 9.**
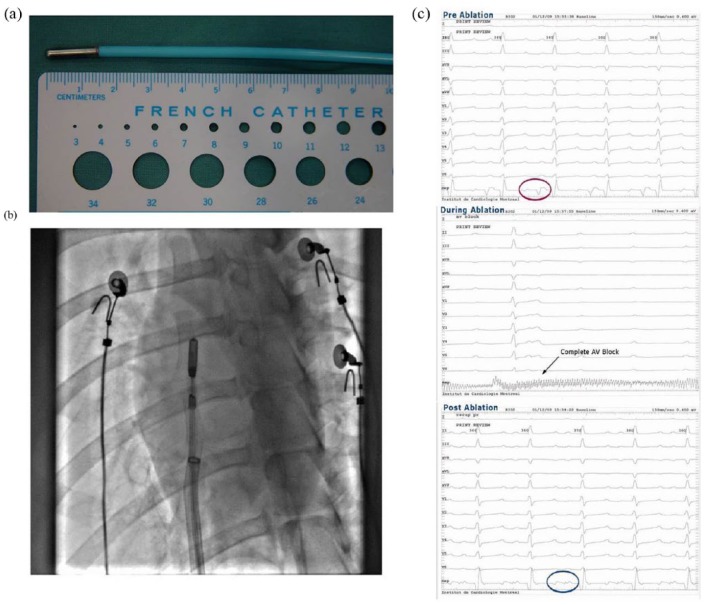
In vivo cryocatheter cardiac tissue ablation: (a) *ICEolate* cryocatheter with a 1.5-cm freeze zone was applied in a canine model to assess the in vivo performance of the SCN cryocatheter ablation system. (b) Canines were utilized and multiple spot and linear lesion sets were created endocardially via catheter introduction through the femoral vein and introduction into the right atria and ventricle. (c) ECG monitoring revealed the complete blockage of electrical conduction following ablation.

**Figure 10.**
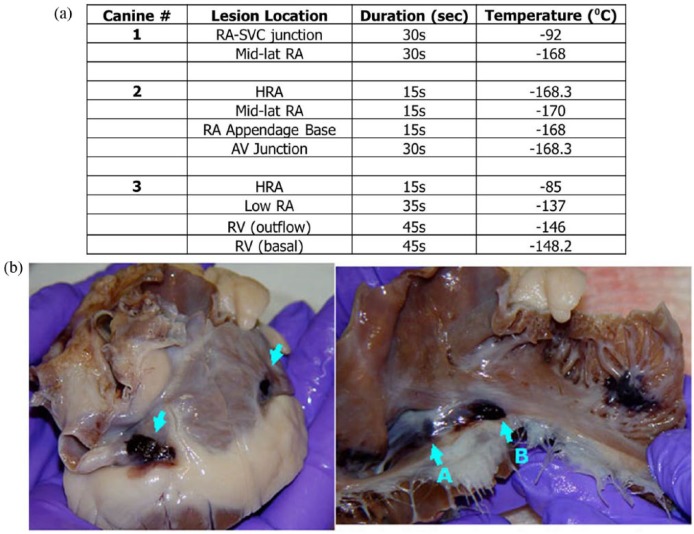
Gross morphological analysis of lesion formation following cryoablation. Following the endocardial freezing procedure, treated hearts were explanted and examined for lesion creation and transmurality. (a) Ablation intervals ranged from 15 to 60 s in various locations in the right atria and ventricle and achieved temperatures ranging from −100°C to −168°C in a fully beating heart depending on the specific time, location, and dosing parameters. (b) Examination of the hearts revealed the creation of complete transmural lesions in the right atria following a 15-s (a) and a 60-s (b) freeze interval.

In addition to ex vivo assessment of *ICEolate* cryocatheter performance, a pilot animal study was also conducted to assess performance in vivo. This study consisted of 10 ablation sites in three mongrel canines wherein the *ICEolate* cryocatheter was inserted into the femoral vein and passed into the right atria and ventricle. CA segment placement within the subject’s heart was accomplished via fluoroscopic visualization (Figure 9(a) and (b)). Once in position, cryogen flow was activated to ablate the target site. A total of 10 ablations were completed in the pilot study (Figure 10(a)). Cardiac electrical activity at each ablation site was monitored prior to, during, and following each cryoablation (Figure 9(c)). In vivo studies revealed the attainment of cryocatheter tip internal temperatures of −168°C within 5 s, resulting in the delivery of an ablative cryogenic “dose” in ~15 to 30 s in atrial tissue, and 45 s with a nadir temperature of −149°C in ventricular tissue (Figure 10(a)). During a 15-s endocardial cryoablation, a temperature of −168°C was achieved at the right atrioventricular (AV) junction tricuspid creating complete AV block during cryoablation (Figures 9 and 10(a)). Gross pathological analysis performed within an hour after freezing revealed that large transmural lesions were created in several areas of the right atria and ventricle as a result of the brief cryo-applications using SCN (Figure 10(b)).

## Discussion

CA is designed to alter cardiac tissue architecture, thereby preventing/correcting cardiac arrhythmias, including VT.^19^ CA strategies have been shown to be effective treatment options.^22–28^ CA is typically performed using radio frequency (RF) to burn cardiac tissue, destroying an arrhythmogenic foci to create a scar resulting in conduction block, thereby preventing propagation of the arrhythmia. Numerous studies have shown CA to be effective.^23–26,29–33^ Patel et al.^30^ reported on a 262 patient study comparing CA to non-ablative strategies and found the rate of recurrent VT was lower in CA populations than in the non-CA cohort. Numerous other reports have demonstrated the efficacy and potential of CA as a first-line strategy for VT.^23,24,26,31–33^ While promising, RF CA is not without issues. Baldinger et al.^29^ recently analyzed the issues with RF CA in a cardiac setting and found some of the reasons for failed or limited ablation success were a result of limited access, failure to identify target, failure to completely ablate the target, proximity to coronary artery and phrenic nerve, among others. It has been suggested that a major cause of recurrent VT following CA is a result of incomplete ablation (creation of a full thickness transmural lesion) and that a combined endo/epicardial ablation approach is often necessary for VT.^32^ While promising, RF CA remains associated with a high degree of recurrence, incidence of damage to adjacent structures (collateral damage), and procedural pain, limiting its widespread use as a first-line treatment.^22,23^

Recently, several studies have suggested that cryoablation is an effective treatment option for VT with fewer complications than RFA.^33–37^ While the use of cryo has been limited for the treatment of VT, it is not without precedent.^33,36–38^ While successful, the application of cryo for ablating VT has been mostly on non-scar related VT as with the current technology, cryo is not very effective on scar-related VT like in ischemic heart disease. The use of cryo for treatment of VT is challenged today by a lack of information on lesion size and thermal dose, effects of vasculature, uniformity of applications, concerns with damage to adjacent tissues, among others.^39–41^ These concerns often hinge upon the inability of today’s Joule–Thomson (JT) argon and nitrous oxide–based cryodevices to deliver an ablative dose through the entire tissue thickness. In practice, today’s commercial cryoablation devices yield nearly equivalent cooling power *at the tip*, attaining temperatures of ~−80°C ≤ 1 mm from the probe surface and have limited heat extraction capacity due to the physics of JT cryogen heat transfer.^21,42–44^ Depending on the distance from the tip, time of application, tissue heat load, uniformity of coverage, and cryogen, actual temperatures attained at the tissue surface and depth of penetration vary significantly.^21,45,46^ These limitations necessitate multiple overlapping, lengthy applications to generate a contiguous lesion while still retaining uncertainty as to the delivery of an “ablative dose.” Issues of patient safety often are discussed related to the extended procedure times and inability to fully freeze cardiac tissue, thereby creating a less-than-optimal treatment paradigm. This has resulted in limiting utilization of cryoablation for the treatment of various cardiac arrhythmias.

In order to overcome these challenges, we have developed a new cryoablation catheter prototype (*ICEolate*) utilizing SCN for the treatment of cardiac arrhythmias. Studies have shown that SCN offers the ability to overcome the limitations of current cryodevices, given that SCN has greater work capacity than JT-based devices as its liquid-like heat extraction properties are more effective than gas.^21,47–49^ This enhanced heat extraction capacity offers the potential to effectively deliver ultra-cold freeze temperatures deep into tissue under high heat load conditions, such as those needed for the successful treatment of VT. If effective, such a technology could provide a new platform enabling the delivery of a precise, minimally invasive cryoablation treatment option for VT, thereby reducing overall procedural outcome variability, cost, recovery time, and associated morbidity. The results presented herein demonstrate that the SCN system in combination with *ICEolate* provides for the rapid delivery of ultra-cold temperatures deep into tissue under high heat loads supporting its potential application for the treatment of various cardiac arrhythmias including VT.

The establishment of ex vivo performance characteristics of new thermal ablation devices, such as cryoablation devices and probes, is important as it provides a reference point for clinical application. While all devices undergo extensive bench and ex vivo testing and characterization prior to pre-clinical and clinical utilization, few ex vivo–based studies have been published detailing the performance of cryoablation devices. The majority of studies published on device performance are focused on animal models or clinical studies and report on findings focusing on a clinical end point of acute, mid-, and long-term outcome. While clinical outcome is clearly important, it is highly dictated by device performance, tissue thickness, tissue contact, final temperature attained throughout the tissue, time of application, time necessary to reach a given temperature and impact of heat load (blood flow), among other things. To this end, the majority of published information pertaining to device performance is restricted to probe temperature (typically measured inside the freeze zone lumen) and time of application. This is unfortunate as it does not provide a body of comparative data for clinicians, researchers, and engineers to draw upon to further understand device performance and thereby limits the ability for optimization of procedures and devices. Furthermore, it makes comparison of current devices as well as the development of new devices challenging.

While presenting a challenge, there are several established parameters which can be utilized for comparison. These performance parameters are dictated by the cryogen utilized. For example, nitrous oxide–based cryodevices can reach a theoretical minimum temperature −89.5°C and argon devices −189°C.^44^ While this is the theoretical coldest temperature that can be attained, studies have shown that the practical minimum working cryoprobe tip temperature which can be obtained within 1 mm of the outer surface of the cryoprobe is ~−80°C or warmer. Studies have also shown that the depths of penetration of the critical −20°C and −40°C isotherms are restricted to the center of the frozen mass (closest to the cryoprobe) comprising less than 35% and 20% of the frozen mass, respectively.^21,44,50,51^ Furthermore, the typical freeze times reported in these and other studies range from 3 to 10 min, which are applied in multiple (repeat) freeze/thaw scenarios. While limited, these data provide a reference point for comparison of new technologies. These data also illustrate the need for the development of new devices which provide for increased cryoablative capacity, which in turn could improve outcome while reducing procedure time.

To this end, we developed a new cryoablation device and cryocatheter (*ICEolate*) which utilizes SCN and is designed to deliver a rapid, controlled ultra-cold ablative dose to a target region. Due to the increased heat capacity of the SCN cryogen compared to argon and nitrous oxide cryogens, SCN also offers the potential to deliver ablative doses deep into tissue while exposed to high heat loads such as experienced in the beating heart.^21,44,47,52^ Given the potential of this system, in this study, we utilized a series of ex vivo bench test models to assess and characterize the performance of this system. Furthermore, these studies were designed to provide a series of bench testing models which could potentially be utilized to provide a reference data set for pre-clinical and clinical study development and protocol optimization. Cryocatheter performance analysis included calorimetry (ablative energy), freeze zone (iceball) size under various heat loads, thermal profiling of the frozen mass in phantom models and the impact of high heat loads (37°C water) in a circulating bath model on lesion formation in an ex vivo tissue model. Calorimetric assessment demonstrated that the *ICEolate* cryocatheter yielded an average cooling power of 126.7 W. Cooling power (wattage) is not typically reported for cryoablation probes and is more commonly reported for hyper thermal ablation such as radio frequency. While not often reported, the limited information available suggests that current argon and nitrous oxide cryoprobes provide in the range of 50–85 W of cooling power. Assessment of freeze zone formation revealed the formation of a ~2-cm-diameter iceball in 3 min or less with the *ICEolate* cryocatheter (Tables 2 and 3 and Figure 5). Isothermal profiling of the frozen mass revealed penetration of the −20°C, −30°C, and −40°C isotherms to a diameter of 2.1, 1.93, and 1.7 cm, respectively, in a 2.3-cm iceball in a 3-min freeze resulting in ~72% of the frozen volume below −20°C, 56% below −30°C, and ~40% below −40°C. This is compared to previous reports on an argon device yielding ~35% and ~20% of the iceball being below −20°C and −40°C, respectively.^21,50^ Furthermore, an independent study on argon probe performance focusing on the −30°C isotherm reported that the probe produced <25% of the frozen mass under −30°C under similar testing conditions.^51^ These data suggest that the SCN *ICEolate* cryocatheter creates a much colder frozen mass compared to several current commercial cryoablation systems. Importantly, in this study, it was found that even under the extremely challenging ex vivo 37°C circulating water bath endocardial tissue model, the *ICEolate* cryocatheter was able to generate lesions with the penetration of the critical −20°C and −30°C isotherms through the full thickness of the tissue model in 45 and 55 s, respectively (Table 4 and Figures 7 and 8). Furthermore, it was found that even when applied to tissue thickness of 1 cm in the 37°C circulating ex vivo endocardial model, *ICEolate* was able to achieve penetration of the −30°C isotherm on the opposite (transmural surface) within 2 min.

Data obtained from the pilot in vivo studies correlated well with the ex vivo findings. Specifically, it was found that the system was able to deliver ultra-cold cryogenic temperatures to the ablation segment in a fully beating heart within a few seconds, and the catheter was able to create a transmural lesion in <1 min (15–45 s) depending on the tissue thickness and cryogen flow rate (Figures 9 and 10). Assessment of electrical activity using ECG revealed the complete blockage of electrical conduction following ablation (Figure 9). The creation of transmural lesions in atrial tissue in 15–30 s in the fully beating hearts in vivo was significantly quicker than that reported for current commercial cryocatheters, which have been reported to necessitate 3- to 5-min freeze interval durations to obtain focal trasmurality in atrial tissue.^53–56^ More notably was the ability for the *ICEolate* to create a transmural lesion in ventricular tissue in the fully beating canine heart in 45–60 s. This was found to be highly promising as the creation of transmural lesions in thicker ventricular tissue is highly challenging with today’s cryodevices and often requires the use of combinatorial strategies to achieve conduction block.^32^

One important observation from this study, beyond that of cryocatheter performance was the fact that outcomes obtained using the SCN *ICEolate* cryocatheter in the pilot in vivo studies correlated well with the ex vivo findings. This is an important observation as it is well appreciated in clinical practice that the heat load of a tissue and cryoprobe proximity to vasculature can impact outcome. The attainment of similar results in both the ex vivo and in vivo studies suggests that the use of ex vivo models provides a means of characterizing and optimizing probe performance and ablative dose delivery, resulting in a better understanding of probe performance and impact on outcome prior to launching in vivo studies. This in turn may provide an avenue for accelerating device development and optimization as well as improved procedure application and clinical outcome.

While the current findings support the potential of the SCN and *ICEolate* technologies, there are several limitations. First, the study was conducted using a large prototype *ICEolate* cryocatheter. The prototype catheter diameter of 10F with a 15-mm ablation segment (electrode) is larger than some commercial cryocatheters which are 7 Fr in diameter with 4–8 mm ablation segments. The larger diameter and ablation segment limited the ability of the catheter to analyze the electrical activity around the ablation site (i.e. reduced resolution). The larger size also resulted in reduced maneuverability of the ablation segment. As the catheter was a prototype, these dimensional limitations were recognized and understood. Despite this limitation, the studies successfully established feasibility of the technology. With this success, current efforts are focused on developing and evaluating a second generation 8 Fr *ICEolate* cryocatheter with several ablation segment sizes, including a 5-mm spot ablation tip and a 15-mm flexible steerable ablation segment. These ablation segments also contain multiple recording electrodes (2 and 4) at 5-mm spacing within the ablation segment. These efforts are focused on providing increased resolution for mapping electrical activity, increased maneuverability of the ablation segment and for increased controllability of ablation zone size.

Another limitation of this study was the pilot nature of the in vivo study. As described, a total of 10 ablations were completed in three subjects to evaluate performance. The pilot study was designed to demonstrate technology feasibility as well as to collect a in vivo data set as a comparative standard for smaller and more maneuverable next generation catheters. The number of subjects and ablations sites was determined using G-Power and designed to minimize the number of subjects while maximizing data collection. The pilot study was also limited to acute analysis of the level of tissue ablation. This prevented in-depth histological examination of the tissue within and surrounding the ablation sites following several days of recovery. Given the accelerated freeze rate provided by the SCN technology, analysis of the ablated cardiac tissue sites in survival studies will be important to demonstrate prolonged precision and long-term efficacy. While additional studies are needed, studies using the SCN technology in other tissue models (renal and prostate) have demonstrated similar lesion characteristic to those created by current commercial devices.^21,44,50^ Although promising, studies are necessary to determine the specific lesion and scar characteristics in cardiac tissue.

## Conclusion

As the use of cryotherapy continues to grow, future improvements to device design and application will further increase its utilization for the treatment of cardiac arrhythmias.^4–13^ With the recent success and growth of cardiac cryoablation, there is now a movement toward the increasing indication utilization and improving procedure outcome. In order to achieve this in an effective, time efficient manner, new cryotechnologies are being developed to facilitate rapid tissue cooling to more effectively treat cardiac arrhythmias. This study investigated the application of SCN-based *ICEolate* cryocatheter for the endovascular cryoablation of cardiac tissue. We hypothesized that through the use of SCN and catheter technologies, that rapid, effective, minimally invasive ablation of cardiac tissue could be achieved. The data from this study suggest that the SCN-based *ICEolate* cryocatheter provides for the rapid and effective delivery of a cryoablative dose within the both the ex vivo and in vivo models evaluated. The data demonstrate that *ICEolate* was able to deliver the necessary “thermal dose” of −30°C^43,53,57^ across tissue ranging from 5 mm to 1 cm in thickness under high heat loads (37°C) in a circulating environment in <60 s. Analysis of the thermal spread of the lethal temperatures during a 2-min freeze was limited to within 5 mm of the probe surface, thereby resulting in the creation of a defined linear ablation zone, a feature which is highly beneficial in the treatment of cardiac arrhythmias. Finally, the in vivo data support our hypothesis and demonstrated that *ICEolate* quickly and controllably created long, contiguous, transmural linear lesions following brief freezing episodes (<60 s). This study arm also confirmed the ability to quickly produce effective ablation of cardiac tissue in both the right atrium and ventricle.

In conclusion, our findings suggest that *ICEolate* offers promise as a next generation transvascular-based cryoablation device for the endocardial based treatment of various cardiac arrhythmias. Our findings also suggest that the *ICEolate* cryocatheter may provide a path for the use of cryoablation in areas which have traditionally challenged cryoablation, such as for the treatment of ventricular arrhythmias. While the results of this study are promising, given their investigational nature the extent of conclusions which can be drawn are limited; however, the data do suggest that this technology holds promise and that continued assessment of the technology in vivo is warranted. Overall, these studies demonstrate that SCN *ICEolate* cryocatheter allows for the rapid and controlled application of ultra-cold temperatures and efficient freezing of targeted cardiac tissue.
